# (*E*)-3-(Anthracen-9-yl)-1-(2-bromo­phen­yl)prop-2-en-1-one

**DOI:** 10.1107/S1600536810048476

**Published:** 2010-11-27

**Authors:** Hoong-Kun Fun, Thawanrat Kobkeatthawin, Jaruwan Joothamongkhon, Suchada Chantrapromma

**Affiliations:** a X-ray Crystallography Unit, School of Physics, Universiti Sains Malaysia, 11800 USM, Penang, Malaysia; b Crystal Materials Research Unit, Department of Chemistry, Faculty of Science, Prince of Songkla University, Hat-Yai, Songkhla 90112, Thailand

## Abstract

The mol­ecule of the title chalcone, C_23_H_15_BrO, is not planar and exists in the *E* configuration with respect to the central C=C bond. The dihedral angle between the benzene and anthracene rings is 83.58 (6)°. The prop-2-en-1-one bridge makes dihedral angles of 63.00 (7) and 42.62 (16)° with the benzene and anthracene rings, respectively. In the crystal, mol­ecules are linked into dimers by weak C—H⋯O inter­actions. These dimers are arranged parallel to the *bc* plane and are further stacked along the *a* axis by π–π inter­actions with a centroid–centroid distance of 3.7561 (9) Å. The crystal structure is further stabilized by C—H⋯π inter­actions.

## Related literature

For bond-length data, see: Allen *et al.* (1987 ▶). For related structures, see: Fun *et al.* (2009 ▶); Joothamongkhon *et al.* (2010 ▶). For background to and applications of chalcones, see: Cheng *et al.* (2008 ▶); Gaber *et al.* (2008 ▶); Joothamongkhon *et al.* (2010 ▶); Nawakowska *et al.* (2008) ▶; Patil & Dharmaprakash (2008 ▶); Tewtrakul *et al.* (2003 ▶). For the stability of the temperature controller used in the data collection, see Cosier & Glazer (1986 ▶).
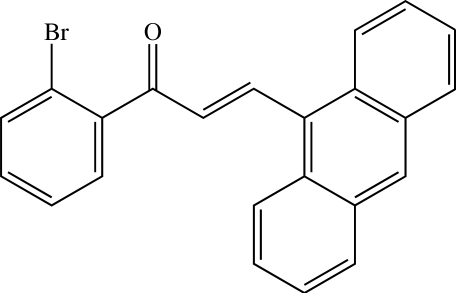


## Experimental

### 

#### Crystal data

C_23_H_15_BrO
*M* *_r_* = 387.25
Orthorhombic, 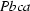

*a* = 7.8631 (1) Å
*b* = 20.0583 (3) Å
*c* = 20.7259 (3) Å
*V* = 3268.90 (8) Å^3^
*Z* = 8
Mo *K*α radiation
μ = 2.52 mm^−1^
*T* = 100 K
0.34 × 0.28 × 0.20 mm

#### Data collection

Bruker APEXII CCD area-detector diffractometer
Absorption correction: multi-scan (*SADABS*; Bruker, 2005 ▶) *T*_min_ = 0.482, *T*_max_ = 0.629
22332 measured reflections
4766 independent reflections
3717 reflections with *I* > 2σ(*I*)
*R*_int_ = 0.038

#### Refinement

*R*[*F* ^2^ > 2σ(*F* ^2^)] = 0.030
*wR*(*F* ^2^) = 0.073
*S* = 1.02
4766 reflections
226 parameters
H-atom parameters constrained
Δρ_max_ = 0.40 e Å^−3^
Δρ_min_ = −0.46 e Å^−3^

### 

Data collection: *APEX2* (Bruker, 2005 ▶); cell refinement: *SAINT* (Bruker, 2005 ▶); data reduction: *SAINT*; program(s) used to solve structure: *SHELXTL* (Sheldrick, 2008 ▶); program(s) used to refine structure: *SHELXTL*; molecular graphics: *SHELXTL*; software used to prepare material for publication: *SHELXTL* and *PLATON* (Spek, 2009 ▶).

## Supplementary Material

Crystal structure: contains datablocks global, I. DOI: 10.1107/S1600536810048476/rz2527sup1.cif

Structure factors: contains datablocks I. DOI: 10.1107/S1600536810048476/rz2527Isup2.hkl

Additional supplementary materials:  crystallographic information; 3D view; checkCIF report

## Figures and Tables

**Table 1:** Hydrogen-bond geometry (Å, °) *Cg*1 is the centroid of the C1–C6 ring.

*D*—H⋯*A*	*D*—H	H⋯*A*	*D*⋯*A*	*D*—H⋯*A*
C5—H5*A*⋯O1^i^	0.93	2.53	3.301 (2)	140
C15—H15*A*⋯*Cg*1^ii^	0.93	2.99	3.6989 (19)	135

Symmetry codes: (i) 
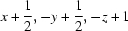
; (ii) 
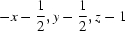
.

**Fig. 1.**
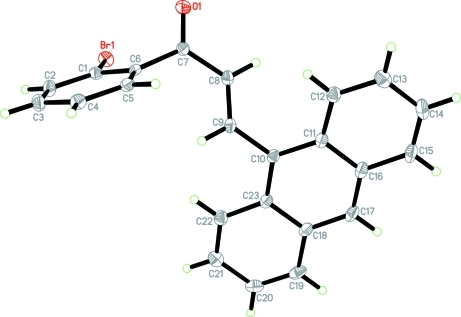
The molecular structure of the title compound, showing 50% probability displacement ellipsoids and the atom-numbering scheme.

**Fig. 2.**
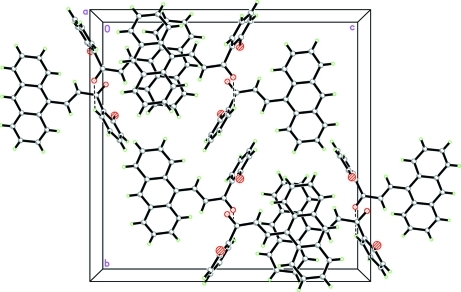
The crystal packing of the title compound viewed along the a axis. C—H···O weak interactions are shown as dashed lines.

## supplementary crystallographic information

### Comment

Chalcones have been studied for their chemical and biological activities for a long time. They have a wide range of applications such as in non-linear optical devices (Patil & Dharmaprakash, 2008) and have various biological properties such as analgesic, anti-inflammatory, antibacterial, antifungal (Nawakowska *et al.*, 2008; Cheng *et al.*, 2008) and HIV-1 protease inhibitory (Tewtrakul *et al.*, 2003) activities. Moreover, chalcones have also been studied for fluorescent property (Gaber *et al.*, 2008). Our previous investigation has revealed that chalcones containing the anthracene moiety displayed fluorescent property (Joothamongkhon *et al.*, 2010). The title compound (I) was synthesized for further investigation of its fluorescent properties. The title compound in chloroform solution exhibited fluorescence with the maximum emission at 450 nm when it was excited at 380 nm.

The molecule of (I) (Fig. 1) exists in an *E* configuration with respect to the C8═C9 double bond [1.343 (2)°], with the torsion angle C7–C8–C9–C10 = 174.24 (16)°. The anthracene unit is essentially planar with the *r.m.s.* 0.0416 (2) Å. The molecule is not planar as indicated by the dihedral angle between benzene and anthracene rings of 83.58 (6)°. The mean plane through the pro-2-en-1-one bridge (C7–C9/O1) [*r.m.s*. 0.0283 (2) Å] makes dihedral angles of 63.00 (7) and 42.62 (16)° with the benzene and anthracene rings, respectively. The bond distances are of normal values (Allen *et al.*, 1987) and are comparable with those of related structures (Fun *et al.*, 2009; Joothamongkhon *et al.*, 2010).

In the crystal packing, the molecules are linked into dimers through the C5—H5A···O1 interactions (Fig. 2). These dimers are arranged into sheets parallel to the *bc* plane, and are further stacked along the *a* axis by π–π interaction with a *Cg*_2_···*Cg*_3_ distance of 3.7561 (9) Å (symmetry code: -1/2 + *x*, *y*, 1/2 - *z*). The crystal structure is further stabilized by C—H···π interactions (Table 1); *Cg*_1_, *Cg*_2_ and *Cg*_3_ are the centroids of the C1–C6, C10–C11/C16–C18/C23 and C11–C16 rings, respectively.

### Experimental

The title compound was synthesized by condensation of 2-bromoacetophenone (0.39 g, 2 mmol) with anthracene-9-carboxaldehyde (0.41 g, 2 mmol) in ethanol (40 ml) in the presence of 20% NaOH (aq) (5 ml). After stirring for 7 h at room temperature, the yellow solid obtained was collected by filtration, washed with distilled water and dried in air. Yellow block-shaped single crystals of the title compound suitable for *x*-ray structure determination were recrystalized from methanol by slow evaporation of the solvent at room temperature after several days. Mp. 427–428 K.

### Refinement

All H atoms were positioned geometrically and allowed to ride on their parent atoms, with d(C—H) = 0.93 Å. The *U*_iso_ values were constrained to be 1.2*U*_eq_ of the carrier atom for all H atoms. The highest residual electron density peak is located at 0.64 Å from C6 and the deepest hole is located at 0.38 Å from Br1.

### Crystal data

**Table:**

C_23_H_15_BrO	*D*_x_ = 1.574 Mg m^−^^3^
*M**_r_* = 387.25	Melting point = 427–428 K
Orthorhombic, *P**b**c**a*	Mo *K*α radiation, λ = 0.71073 Å
Hall symbol: -P 2ac 2ab	Cell parameters from 4766 reflections
*a* = 7.8631 (1) Å	θ = 2.0–30.0°
*b* = 20.0583 (3) Å	µ = 2.52 mm^−^^1^
*c* = 20.7259 (3) Å	*T* = 100 K
*V* = 3268.90 (8) Å^3^	Block, yellow
*Z* = 8	0.34 × 0.28 × 0.20 mm
*F*(000) = 1568	

### Data collection

**Table:**

Bruker APEXII CCD area-detector diffractometer	4766 independent reflections
Radiation source: sealed tube	3717 reflections with *I* > 2σ(*I*)
graphite	*R*_int_ = 0.038
φ and ω scans	θ_max_ = 30.0°, θ_min_ = 2.0°
Absorption correction: multi-scan (*SADABS*; Bruker, 2005)	*h* = −11→9
*T*_min_ = 0.482, *T*_max_ = 0.629	*k* = −28→21
22332 measured reflections	*l* = −26→29

### Refinement

**Table:**

Refinement on *F*^2^	Primary atom site location: structure-invariant direct methods
Least-squares matrix: full	Secondary atom site location: difference Fourier map
*R*[*F*^2^ > 2σ(*F*^2^)] = 0.030	Hydrogen site location: inferred from neighbouring sites
*wR*(*F*^2^) = 0.073	H-atom parameters constrained
*S* = 1.02	*w* = 1/[σ^2^(*F*_o_^2^) + (0.0336*P*)^2^ + 1.3057*P*] where *P* = (*F*_o_^2^ + 2*F*_c_^2^)/3
4766 reflections	(Δ/σ)_max_ = 0.002
226 parameters	Δρ_max_ = 0.40 e Å^−^^3^
0 restraints	Δρ_min_ = −0.46 e Å^−^^3^

### Special details

**Table:**

Experimental. The crystal was placed in the cold stream of an Oxford Cryosystems Cobra open-flow nitrogen cryostat (Cosier & Glazer, 1986) operating at 120.0 (1) K.
Geometry. All e.s.d.'s (except the e.s.d. in the dihedral angle between two l.s. planes) are estimated using the full covariance matrix. The cell e.s.d.'s are taken into account individually in the estimation of e.s.d.'s in distances, angles and torsion angles; correlations between e.s.d.'s in cell parameters are only used when they are defined by crystal symmetry. An approximate (isotropic) treatment of cell e.s.d.'s is used for estimating e.s.d.'s involving l.s. planes.
Refinement. Refinement of *F*^2^ against ALL reflections. The weighted *R*-factor *wR* and goodness of fit *S* are based on *F*^2^, conventional *R*-factors *R* are based on *F*, with *F* set to zero for negative *F*^2^. The threshold expression of *F*^2^ > σ(*F*^2^) is used only for calculating *R*-factors(gt) *etc*. and is not relevant to the choice of reflections for refinement. *R*-factors based on *F*^2^ are statistically about twice as large as those based on *F*, and *R*- factors based on ALL data will be even larger.

### Fractional atomic coordinates and isotropic or equivalent isotropic displacement parameters (Å^2^)

**Table:**

	*x*	*y*	*z*	*U*_iso_*/*U*_eq_	
Br1	−0.28035 (2)	0.124729 (8)	0.534873 (8)	0.01848 (6)	
O1	−0.02804 (17)	0.24813 (6)	0.51069 (6)	0.0207 (3)	
C1	−0.0478 (2)	0.09958 (8)	0.53816 (8)	0.0141 (3)	
C2	−0.0051 (2)	0.04186 (8)	0.57118 (8)	0.0182 (3)	
H2A	−0.0883	0.0173	0.5925	0.022*	
C3	0.1634 (2)	0.02107 (9)	0.57209 (8)	0.0187 (3)	
H3A	0.1936	−0.0173	0.5946	0.022*	
C4	0.2868 (2)	0.05739 (9)	0.53953 (8)	0.0169 (3)	
H4A	0.3991	0.0429	0.5396	0.020*	
C5	0.2427 (2)	0.11522 (8)	0.50691 (8)	0.0148 (3)	
H5A	0.3260	0.1393	0.4852	0.018*	
C6	0.0743 (2)	0.13775 (8)	0.50626 (7)	0.0130 (3)	
C7	0.0308 (2)	0.20401 (8)	0.47631 (7)	0.0144 (3)	
C8	0.0571 (2)	0.21502 (8)	0.40715 (8)	0.0152 (3)	
H8A	0.0380	0.2577	0.3913	0.018*	
C9	0.1067 (2)	0.16805 (8)	0.36499 (8)	0.0143 (3)	
H9A	0.1357	0.1266	0.3817	0.017*	
C10	0.1193 (2)	0.17648 (8)	0.29459 (7)	0.0136 (3)	
C11	0.1861 (2)	0.23539 (8)	0.26620 (8)	0.0146 (3)	
C12	0.2644 (2)	0.28787 (8)	0.30234 (9)	0.0172 (3)	
H12A	0.2765	0.2832	0.3467	0.021*	
C13	0.3216 (2)	0.34451 (9)	0.27314 (9)	0.0210 (4)	
H13A	0.3715	0.3779	0.2978	0.025*	
C14	0.3059 (2)	0.35296 (9)	0.20539 (9)	0.0227 (4)	
H14A	0.3417	0.3924	0.1861	0.027*	
C15	0.2388 (2)	0.30366 (9)	0.16882 (9)	0.0211 (4)	
H15A	0.2303	0.3095	0.1244	0.025*	
C16	0.1807 (2)	0.24275 (8)	0.19713 (8)	0.0168 (3)	
C17	0.1193 (2)	0.19111 (8)	0.15893 (8)	0.0184 (3)	
H17A	0.1169	0.1964	0.1144	0.022*	
C18	0.0612 (2)	0.13157 (8)	0.18586 (8)	0.0161 (3)	
C19	0.0007 (2)	0.07806 (9)	0.14641 (8)	0.0206 (4)	
H19A	0.0033	0.0825	0.1018	0.025*	
C20	−0.0605 (2)	0.02092 (9)	0.17272 (9)	0.0213 (4)	
H20A	−0.0984	−0.0134	0.1462	0.026*	
C21	−0.0666 (2)	0.01375 (9)	0.24085 (9)	0.0208 (4)	
H21A	−0.1095	−0.0252	0.2589	0.025*	
C22	−0.0101 (2)	0.06361 (8)	0.28000 (8)	0.0178 (3)	
H22A	−0.0167	0.0581	0.3245	0.021*	
C23	0.0591 (2)	0.12412 (8)	0.25471 (8)	0.0143 (3)	

### Atomic displacement parameters (Å^2^)

**Table:**

	*U*^11^	*U*^22^	*U*^33^	*U*^12^	*U*^13^	*U*^23^
Br1	0.01349 (9)	0.01896 (9)	0.02300 (10)	0.00166 (6)	0.00026 (7)	0.00057 (7)
O1	0.0275 (7)	0.0153 (6)	0.0193 (6)	0.0025 (5)	0.0052 (5)	−0.0013 (5)
C1	0.0132 (8)	0.0144 (7)	0.0146 (8)	0.0010 (6)	−0.0003 (6)	−0.0014 (6)
C2	0.0190 (9)	0.0186 (8)	0.0168 (8)	−0.0012 (7)	0.0029 (7)	0.0039 (6)
C3	0.0209 (9)	0.0175 (8)	0.0175 (8)	0.0021 (7)	−0.0002 (7)	0.0039 (6)
C4	0.0156 (8)	0.0187 (8)	0.0165 (8)	0.0013 (6)	−0.0001 (7)	−0.0009 (6)
C5	0.0171 (9)	0.0150 (8)	0.0124 (7)	−0.0016 (6)	0.0015 (6)	−0.0012 (6)
C6	0.0166 (8)	0.0130 (7)	0.0094 (7)	−0.0002 (6)	−0.0005 (6)	−0.0015 (5)
C7	0.0149 (8)	0.0136 (7)	0.0147 (8)	−0.0022 (6)	0.0004 (6)	0.0003 (6)
C8	0.0164 (8)	0.0145 (8)	0.0147 (8)	0.0008 (6)	−0.0001 (6)	0.0030 (6)
C9	0.0150 (8)	0.0148 (7)	0.0131 (7)	−0.0006 (6)	−0.0012 (6)	0.0036 (6)
C10	0.0139 (8)	0.0152 (8)	0.0117 (7)	0.0030 (6)	0.0006 (6)	0.0015 (6)
C11	0.0128 (8)	0.0163 (8)	0.0148 (8)	0.0038 (6)	0.0016 (6)	0.0031 (6)
C12	0.0169 (8)	0.0178 (8)	0.0170 (8)	0.0024 (6)	0.0024 (6)	0.0021 (6)
C13	0.0168 (9)	0.0181 (9)	0.0280 (9)	−0.0003 (7)	0.0030 (7)	0.0005 (7)
C14	0.0196 (10)	0.0196 (8)	0.0290 (10)	0.0019 (7)	0.0065 (7)	0.0099 (7)
C15	0.0213 (9)	0.0243 (9)	0.0178 (8)	0.0060 (7)	0.0052 (7)	0.0097 (7)
C16	0.0144 (8)	0.0201 (8)	0.0159 (8)	0.0049 (6)	0.0028 (6)	0.0054 (6)
C17	0.0183 (9)	0.0248 (9)	0.0122 (8)	0.0071 (7)	0.0022 (6)	0.0044 (6)
C18	0.0158 (8)	0.0201 (8)	0.0122 (7)	0.0047 (6)	0.0007 (6)	0.0007 (6)
C19	0.0201 (9)	0.0283 (9)	0.0134 (8)	0.0065 (7)	−0.0030 (7)	−0.0044 (7)
C20	0.0207 (9)	0.0220 (8)	0.0212 (9)	0.0038 (7)	−0.0029 (7)	−0.0074 (7)
C21	0.0179 (9)	0.0191 (8)	0.0254 (9)	−0.0002 (7)	0.0002 (7)	0.0004 (7)
C22	0.0179 (9)	0.0185 (8)	0.0169 (8)	0.0015 (6)	0.0001 (6)	0.0021 (6)
C23	0.0130 (8)	0.0170 (8)	0.0128 (7)	0.0045 (6)	−0.0001 (6)	0.0019 (6)

### Geometric parameters (Å, °)

**Table:**

Br1—C1	1.8983 (17)	C12—C13	1.364 (2)
O1—C7	1.2269 (19)	C12—H12A	0.9300
C1—C2	1.386 (2)	C13—C14	1.420 (3)
C1—C6	1.395 (2)	C13—H13A	0.9300
C2—C3	1.389 (2)	C14—C15	1.353 (3)
C2—H2A	0.9300	C14—H14A	0.9300
C3—C4	1.388 (2)	C15—C16	1.430 (2)
C3—H3A	0.9300	C15—H15A	0.9300
C4—C5	1.387 (2)	C16—C17	1.390 (2)
C4—H4A	0.9300	C17—C18	1.395 (2)
C5—C6	1.399 (2)	C17—H17A	0.9300
C5—H5A	0.9300	C18—C19	1.431 (2)
C6—C7	1.506 (2)	C18—C23	1.435 (2)
C7—C8	1.465 (2)	C19—C20	1.357 (3)
C8—C9	1.343 (2)	C19—H19A	0.9300
C8—H8A	0.9300	C20—C21	1.420 (2)
C9—C10	1.472 (2)	C20—H20A	0.9300
C9—H9A	0.9300	C21—C22	1.362 (2)
C10—C23	1.418 (2)	C21—H21A	0.9300
C10—C11	1.421 (2)	C22—C23	1.430 (2)
C11—C12	1.431 (2)	C22—H22A	0.9300
C11—C16	1.440 (2)		
			
C2—C1—C6	121.74 (16)	C11—C12—H12A	119.3
C2—C1—Br1	118.21 (13)	C12—C13—C14	120.64 (17)
C6—C1—Br1	120.02 (12)	C12—C13—H13A	119.7
C1—C2—C3	119.22 (16)	C14—C13—H13A	119.7
C1—C2—H2A	120.4	C15—C14—C13	120.04 (16)
C3—C2—H2A	120.4	C15—C14—H14A	120.0
C4—C3—C2	120.15 (16)	C13—C14—H14A	120.0
C4—C3—H3A	119.9	C14—C15—C16	121.28 (17)
C2—C3—H3A	119.9	C14—C15—H15A	119.4
C5—C4—C3	120.10 (16)	C16—C15—H15A	119.4
C5—C4—H4A	120.0	C17—C16—C15	120.92 (16)
C3—C4—H4A	120.0	C17—C16—C11	120.00 (15)
C4—C5—C6	120.76 (15)	C15—C16—C11	119.08 (16)
C4—C5—H5A	119.6	C16—C17—C18	121.58 (15)
C6—C5—H5A	119.6	C16—C17—H17A	119.2
C1—C6—C5	118.00 (15)	C18—C17—H17A	119.2
C1—C6—C7	121.57 (15)	C17—C18—C19	121.51 (15)
C5—C6—C7	120.25 (15)	C17—C18—C23	119.39 (15)
O1—C7—C8	120.84 (14)	C19—C18—C23	119.09 (15)
O1—C7—C6	118.86 (14)	C20—C19—C18	121.46 (16)
C8—C7—C6	120.29 (14)	C20—C19—H19A	119.3
C9—C8—C7	124.87 (15)	C18—C19—H19A	119.3
C9—C8—H8A	117.6	C19—C20—C21	119.79 (16)
C7—C8—H8A	117.6	C19—C20—H20A	120.1
C8—C9—C10	125.72 (15)	C21—C20—H20A	120.1
C8—C9—H9A	117.1	C22—C21—C20	120.45 (17)
C10—C9—H9A	117.1	C22—C21—H21A	119.8
C23—C10—C11	119.88 (14)	C20—C21—H21A	119.8
C23—C10—C9	118.06 (14)	C21—C22—C23	121.94 (16)
C11—C10—C9	122.05 (14)	C21—C22—H22A	119.0
C10—C11—C12	123.65 (15)	C23—C22—H22A	119.0
C10—C11—C16	119.09 (15)	C10—C23—C22	122.83 (15)
C12—C11—C16	117.23 (15)	C10—C23—C18	119.92 (14)
C13—C12—C11	121.50 (16)	C22—C23—C18	117.22 (15)
C13—C12—H12A	119.3		
			
C6—C1—C2—C3	−0.6 (3)	C12—C13—C14—C15	−2.3 (3)
Br1—C1—C2—C3	177.50 (13)	C13—C14—C15—C16	0.8 (3)
C1—C2—C3—C4	−0.8 (3)	C14—C15—C16—C17	−176.97 (17)
C2—C3—C4—C5	1.1 (3)	C14—C15—C16—C11	3.2 (3)
C3—C4—C5—C6	0.0 (2)	C10—C11—C16—C17	−3.5 (2)
C2—C1—C6—C5	1.7 (2)	C12—C11—C16—C17	174.62 (15)
Br1—C1—C6—C5	−176.39 (12)	C10—C11—C16—C15	176.38 (15)
C2—C1—C6—C7	−173.58 (15)	C12—C11—C16—C15	−5.5 (2)
Br1—C1—C6—C7	8.4 (2)	C15—C16—C17—C18	−179.59 (16)
C4—C5—C6—C1	−1.4 (2)	C11—C16—C17—C18	0.2 (3)
C4—C5—C6—C7	173.95 (15)	C16—C17—C18—C19	−178.97 (16)
C1—C6—C7—O1	58.3 (2)	C16—C17—C18—C23	2.2 (3)
C5—C6—C7—O1	−116.82 (18)	C17—C18—C19—C20	−178.01 (17)
C1—C6—C7—C8	−121.07 (17)	C23—C18—C19—C20	0.8 (3)
C5—C6—C7—C8	63.8 (2)	C18—C19—C20—C21	0.6 (3)
O1—C7—C8—C9	−173.96 (17)	C19—C20—C21—C22	−0.6 (3)
C6—C7—C8—C9	5.4 (3)	C20—C21—C22—C23	−0.9 (3)
C7—C8—C9—C10	174.24 (16)	C11—C10—C23—C22	−179.81 (15)
C8—C9—C10—C23	−137.39 (17)	C9—C10—C23—C22	−1.0 (2)
C8—C9—C10—C11	41.4 (3)	C11—C10—C23—C18	−1.8 (2)
C23—C10—C11—C12	−173.75 (15)	C9—C10—C23—C18	176.97 (15)
C9—C10—C11—C12	7.5 (2)	C21—C22—C23—C10	−179.72 (16)
C23—C10—C11—C16	4.2 (2)	C21—C22—C23—C18	2.2 (2)
C9—C10—C11—C16	−174.54 (15)	C17—C18—C23—C10	−1.4 (2)
C10—C11—C12—C13	−177.81 (16)	C19—C18—C23—C10	179.73 (15)
C16—C11—C12—C13	4.2 (2)	C17—C18—C23—C22	176.71 (15)
C11—C12—C13—C14	−0.4 (3)	C19—C18—C23—C22	−2.2 (2)

### Hydrogen-bond geometry (Å, °)

**Table:**

Cg1 is the centroid of the C1–C6 ring.

**Table:**

*D*—H···*A*	*D*—H	H···*A*	*D*···*A*	*D*—H···*A*
C5—H5A···O1^i^	0.93	2.53	3.301 (2)	140
C15—H15A···Cg1^ii^	0.93	2.99	3.6989 (19)	135

Symmetry codes: (i) *x*+1/2, −*y*+1/2, −*z*+1; (ii) −*x*−1/2, *y*−1/2, *z*−1.
